# Use of Artificial Intelligence in Public Health Education for Pandemic Preparedness and Response

**DOI:** 10.5334/aogh.5130

**Published:** 2026-02-20

**Authors:** Ellen Crystian Silvestre Garcia Souza, Aires Garcia dos Santos Junior, Adriana M. S. Félix, João Paulo Assunção Borges, Layze Braz de Oliveira, Liliane Moretti Carneiro, Alvaro Francisco Lopes de Sousa

**Affiliations:** 1 Universidade Federal de Mato Grosso do Sul (UFMS), Três Lagoas Campus, MS, 79600-080, Brazil; 2 School of Nursing, Universidade de São Paulo, São Paulo, SP, 05403-000, Brazil; 3 Universidade Federal Mato Grosso do Sul (UFMS), Coxim Campus, MS, 79400-000, Brazil; 4 Universidade Federal de Maran˙hão, São Luís 65080-805 Maranhao, Brazil

**Keywords:** artificial intelligence, public health education, pandemics, chatbots, machine learning, misinformation

## Abstract

*Background:* The rapid evolution of artificial intelligence (AI) has enabled new approaches for health education, particularly during public health emergencies. However, evidence remains fragmented on how AI-based educational strategies support preparedness, response, and recovery phases of pandemics and epidemics.

*Objective:* To map the use of AI-based technologies in health education strategies addressing preparedness, response, and recovery during public health emergencies, identifying target populations, intervention characteristics, outcomes, scalability, and knowledge gaps.

*Methods:* This scoping review followed Joanna Briggs Institute methodology and PRISMA-ScR guidelines. Searches were conducted in PubMed/MEDLINE, Scopus, Web of Science, Embase, IEEE Xplore, and LILACS, complemented by gray literature from Google Scholar. Studies published from 2010 onward in English, Portuguese, or Spanish were included. Eligible designs comprised primary studies, methodological or implementation research, and reviews with explicit educational components. Data extraction covered context, populations, AI modalities, educational purposes, delivery channels, supervision requirements, pandemic-cycle phase, scalability, outcomes, and evidence gaps.

*Results:* Forty-one studies met the inclusion criteria. Conversational AI (chatbots and large language models) and algorithmic curation tools using machine learning and natural language processing predominated. Most interventions supported health literacy, risk communication, and misinformation management; others addressed personalized learning, microtraining, and clinical simulation for students and health professionals. Delivery channels included mobile applications, messaging platforms, websites/YouTube, and clinical AI systems. Human oversight (expert validation and curation) was consistently reported as essential for safety and reliability. Interventions mainly targeted the response phase, with emerging applications for preparedness. Major gaps included standardized learning measures, cost-effectiveness evaluations, equity analyses, and governance frameworks ensuring privacy, transparency, and bias control.

*Conclusions:* AI-enabled educational technologies can strengthen rapid, scalable, and personalized learning during health emergencies. Future research should prioritize multicenter studies using standardized indicators, economic and equity assessments, and robust governance frameworks to ensure ethical, safe, and inclusive adoption.

## Introduction

Public health emergencies experienced in recent decades, including the COVID-19 pandemic and the monkeypox outbreak, as well as earlier events such as SARS (2003), H1N1 influenza, Ebola, Zika, and the reemergence of poliomyelitis, all classified by the World Health Organization (WHO) as Public Health Emergencies of International Concern (PHEICs), have forcefully exposed the vulnerability of health systems [1]. These critical scenarios have brought to light major structural and operational weaknesses in health systems, especially in epidemiological surveillance and in their capacity to prepare for, respond to, and recover efficiently from health crises. In addition, they have revealed gaps in professional training, health communication, and institutional agility, underscoring the urgency of more resilient, intersectoral, and integrated systems [1].

In this context, public health education plays a strategic role by strengthening the capacity for early risk identification, situational analysis, and decision-making in the face of emergencies, integrating technical, scientific, and technological competencies focused on prevention, harm mitigation, surveillance, and rapid response [1]. Among the innovations driving this transformation, artificial intelligence (AI) stands out, with applications ranging from epidemiological modeling and outbreak forecasting to clinical decision support and the personalization of educational processes [1, 2].

During the COVID-19 pandemic, AI-based solutions, such as adaptive platforms, intelligent tutors, chatbots, and recommender systems, made it possible to maintain professional training while expanding access to health information, even in the context of mobility restrictions and physical distancing [1, 2]. This integration between AI and education has proven essential for preparing professionals to work in complex scenarios characterized by high uncertainty, large data volumes, and the need for rapid, evidence-based decisions [3]. Consistent with this perspective, the WHO emphasized in a 2018 report that digital technologies and AI are central instruments for achieving global health goals, such as expanding universal coverage, protecting populations from emergencies, and promoting the well-being of billions of people [4].

In emergency management, AI has helped improve real-time monitoring, strengthen intersectoral coordination, optimize resources, and build the capacity of response teams [5]. At the same time, intelligent systems have expanded post-pandemic surveillance capacity, enabling early threat detection and the identification of emerging epidemiological patterns [6]. Despite this transformative potential, ethical and structural challenges remain, related to algorithmic bias, data privacy, and inequalities in access to digital infrastructure [1, 3].

Given this scenario, integrating AI into educational strategies constitutes an emerging and promising frontier in public health, with the potential to enhance professional training and performance during health crises. However, syntheses that consolidate the evidence on this interface are still scarce. Thus, this scoping review aims to map the evidence on the use of AI-based technologies in educational strategies targeting the preparedness, response, and recovery phases of public health emergencies, including pandemics, epidemics, and other events, by describing interventions, target populations, technologies, outcomes, and knowledge gaps.

## Methods

### Type of study

This research was conducted as a scoping review, following the methodological framework proposed by Arksey and O’Malley and further developed by the Joanna Briggs Institute (JBI) [7]. The protocol was structured to ensure transparency and reproducibility of the process, following the steps: (1) identification of the research question; (2) identification of relevant studies; (3) study selection; (4) data extraction; and (5) collection, synthesis, and reporting of results.

Accordingly, the research question and key search elements for this review were developed using the PCC strategy, a mnemonic that helps identify the core topics: problem, concept, and context. In this review, the problem was defined as the use of AI-based technologies; the central concept was public health educational strategies; and the context was the preparedness, response, and recovery phases in the setting of pandemics, epidemics, and other emergencies. Thus, the guiding research question was: What scientific evidence exists on the use of AI-based technologies in educational strategies targeting the preparedness, response, and recovery phases of public health emergencies, including pandemics, epidemics, and other events of public health relevance?

### Study selection

The articles were stored and organized in the Zotero© reference manager. For study selection, search results were independently screened by two researchers using Google Forms© and Google Sheets©. Discrepancies were resolved by consensus or with the involvement of a third researcher for adjudication. The researchers compared the results of their searches, verified differences in findings, and consistently sought to include the largest possible number of eligible studies.

### Search and data collection

Searches were performed in major bibliographic databases, PubMed/MEDLINE, Scopus, Web of Science, Embase, IEEE Xplore, and LILACS, and complemented by a targeted search of gray literature in Google Scholar, limited to the first pages ranked by relevance, as well as institutional documents cited in the included studies, such as those from the World Health Organization (WHO), the Pan American Health Organization (PAHO), and the Centers for Disease Control and Prevention (CDC). The strategies combined controlled descriptors and free-text terms, tailored to each database, with Boolean operators, truncation, and restriction to title, abstract, and subject fields. Filters were applied for the period from 2010 onward and for publications in English, Portuguese, and Spanish. Complete search strategies for each database were archived to support reproducibility, and searches were last updated in October 2025.

Eligible studies included primary quantitative, qualitative, or mixed-methods research, as well as methodological studies and implementation reports that described the explicit use of AI for educational purposes in public health in either real or simulated pandemic scenarios. Narrative and integrative reviews and bibliometric studies were also included when they provided syntheses applicable to educational practice. We excluded reports without an educational component (for example, purely technical surveillance or diagnostic applications), editorials and opinion pieces without methodological or implementation contributions, studies without full-text access, and publications in languages other than English, Portuguese, and Spanish.

Record management included export in RIS and BibTeX formats and integration into a bibliographic manager, followed by two-step deduplication: an automated software process (based on title, authors, year, and DOI) and manual inspection for residual cases. Study selection was conducted in two phases by two independent reviewers: title and abstract screening based on eligibility criteria and full-text assessment of potentially relevant records. Disagreements were resolved by consensus and, when necessary, by a third reviewer. Records without full-text access, after attempts to contact authors or institutions, were excluded with appropriate justification. The eligibility flow was documented in a PRISMA-ScR diagram.

Data extraction was performed using a pilot-tested form that captured: identification (author, year, country), public health emergency addressed, target population and channel/setting, AI modality and educational purpose, platform and presence of human curation/supervision, phase of the pandemic cycle, operational and scalability aspects, study type, and main reported limitations. When available, we also recorded educational outcomes, engagement and acceptability metrics, references to equity and language, costs/implementation, and ethical considerations. Missing information was explicitly classified as “not reported (NR),” avoiding unsupported imputations.

The synthesis combined descriptive and narrative approaches, organized into three complementary tables: (i) characterization of studies (identification, context, population, and design); (ii) AI interventions and strategies with their educational objectives, channels, and supervision; and (iii) functional synthesis linking the educational role of each intervention to pandemic cycle phases, AI-mediated pedagogical mechanisms, scalability/operability conditions, level of evidence, and key gaps. The interpretive narrative integrated convergent findings and contextual variations, as well as recurrent absences of standardized metrics for educational and behavioral effects, economic evaluations, and equity analyses.

Given the inherent scope of mapping reviews, we did not conduct a formal risk-of-bias assessment. To strengthen interpretability, we explicitly reported the study type and limitations declared by the authors (for example, restricted samples, dependence on specific platforms, language bias, and absence of clinical outcomes) and discussed their impact on the generalizability and applicability of the findings. This strategy supports methodological transparency and coherence between the aim of mapping the field and the level of inference that is appropriate based on the available body of evidence.

## Results

Table 1 presents a synthesis of 31 studies published between 2020 and 2025, encompassing different methodological designs, ranging from narrative and integrative reviews to observational and experimental studies. A predominance of exploratory research and narrative reviews was observed, highlighting the still-emerging and consolidating field of AI in educational strategies applied to public health. Most studies were conducted in the context of the COVID-19 pandemic, although some initiatives also addressed other epidemic situations or post-pandemic phases, with an emphasis on surveillance, risk communication, and training of health professionals.

**Table 1:** Methodological and contextual characteristics of the studies included in the review (*n* = 31), Brazil, 2020–2025.

ID (AUTHOR, YEAR)	TYPE	AI/EDUCATIONAL STRATEGY	AUDIENCE/CHANNEL	MAIN FINDINGS	KEY LIMITATION
Guo et al. (2024) [8]	Methodological study (ML)	Algorithmic curation of videos (YouTube); proposal of chatbots and integration into apps	Public/professionals; YouTube/apps	Improves the quality and discovery of trustworthy content; feasible integration with official channels	No behavioral evaluation in real-world settings; platform dependence
Xie et al. (2024) [9]	Integrative review	AI chatbots for education/clinical tutoring	Students/educators; digital environment	Potential for personalized tutoring and post-pandemic support	Heterogeneous evidence; no clinical outcomes
Franchini et al. (2021) [10]	Implementation study (mixed methods)	Community chatbot (Dress-COV) for triage/education	Adults (Telegram)	Reach and interaction with participatory education	Nonequivalent control; limited generalizability
Văduva et al. (2023) [11]	Narrative review	eHealth/mHealth/telemedicine (includes AI)	Hospital nurses	Expands access and remote training	Non-systematic; no effect metrics
Parums (2021) [12]	Editorial	Digital transformation (includes AI)	—	Emphasizes the educational role of digital health	No empirical data
Abdelouahed et al. (2025) [1]	Exploratory qualitative study	Adaptive AI; simulators; personalized content	Professionals/managers	Continuous training and tailored materials	Documentary/case-based; no measurement
Tekinay (2023) [13]	Exploratory study	ChatGPT as educator	Public questions (COVID-19)	Accessible and rapid responses	Qualitative assessment; LLM biases
Sezgin and Kocaballi (2025) [14]	Exploratory study	Generative AI in messaging (WhatsApp/SMS)	Frequently asked public health questions	Greater clarity and accuracy of responses	No behavioral outcomes
McKee et al. (2025) [15]	Applied narrative review	Data/AI for segmented communication	Public health professionals	Popular and digital education with greater impact	Non-systematic
Haupt et al. (2024) [16]	Experimental study (prompts)	Media literacy/AI (role-playing game versus neutral)	Users/trainees	Better misinformation detection with appropriate prompting	Limited sample/scope
Tanui et al. (2024) [17]	Narrative review	Apps with multilingual AI	African populations (general)	Inclusive and scalable education	Descriptive evidence
Bharel et al. (2024) [18]	Perspective	Generative AI for communication/efficiency	Health professionals/organizations	Reduces administrative burden; supports messaging	No empirical data
Meo et al. (2023) [19]	Performance evaluation	ChatGPT (health questions)	—	Good performance on educational FAQs	No link to behavior
Zeeb et al. (2023) [20]	Descriptive narrative	Apps/digital platforms	Population of Bremen	Awareness via apps during COVID-19	No causal evaluation
Towler et al. (2023) [21]	Methodological study	ML (topic modeling) for rapid analysis of qualitative data	COVID-19 textual data	Accelerates insights for communication	Does not measure public impact
Ma et al. (2023) [22]	Cross-sectional study	Digital health curriculum with AI	Health students (China)	Need for curricular integration and practice	Self-reported; non-experimental
Jia et al. (2023) [23]	Narrative review	Training in surveillance with AI	Professionals/public	Training plus real-time alerts	No educational measurement
He et al. (2022) [24]	Observational study	AI in diagnosis/CT (with educational pathway)	Professionals	Training for clinical AI use	Clinical focus; indirect education
Grüne et al. (2022) [25]	Retrospective observational study	Symptom app with feedback	App users	Self-care and awareness	Use bias; no counterfactual
Weeks et al. (2022) [26]	Qualitative study	Personalized chatbot for vaccine hesitancy	Urban youth	Empathic messages increase acceptance	Qualitative; no population-level effect
Dzau et al. (2022) [27]	Narrative review	Digital capacity-building frameworks	Professionals/students	Proposes simulations and remote teaching	No impact data
Wang et al. (2023) [2]	Narrative review/conceptual paper	AI-enhanced curriculum; data-driven teaching	Public health students/educators; university courses	Framework to integrate AI and big data into public health education	Conceptual paper; no empirical evaluation
Wen et al. (2023) [28]	Bibliometric study	Trends in digital/AI research	—	Identifies frontiers (social media)	No educational outcomes
Wang and Li (2024) [3]	Narrative review/perspective	Adaptive learning; AI tutoring; simulations	Public health/medical students and professionals;	Digital platforms/simulation-based training AI can personalize learning and support simulation-based public health training at scale	Theoretical overview; no primary data or implementation studies
Scott and Coiera (2020) [29]	Critical narrative review	Early warning/NLP and modeling	Patients/professionals	Supports policies and messaging	No direct educational assessment
Uohara et al. (2020) [30]	Narrative review	Triage chatbots; telemonitoring	Professionals/public	Scales recommendations and recruitment	No trials
Montenegro-López (2020) [31]	Descriptive study	National app plus AI committee	Professionals/patients	Guidance and local management	Qualitative/documentary
Simsek and Kantarci (2020) [32]	Case/modeling study	Optimized allocation (AI)	Managers	Informs planning/education	No direct educational channel
McKillop et al. (2021) [33]	Mixed-methods exploratory study	COVID-19 chatbots based on CDC/WHO	Citizens	Positive use and acceptability	Uncertain behavioral effect
Verma et al. (2025) [34]	Feasibility study (mixed methods)	Hospital educational technology	Visitors/patients (OPD)	Improved compliance during the intervention	Single-center; short term
Bynon Neely et al. (2024) [35]	Exploratory study	YouTube plus SEO with ChatGPT support	Communities and health workers	Engagement and reach of videos	No causal evaluation

The most frequently used AI technologies included chatbots, adaptive learning platforms, algorithmic curation systems, and machine learning (ML) models focused on the analysis, synthesis, and dissemination of information. These solutions were applied both in formal educational settings, such as universities, continuing education programs, and clinical training, and in public communication strategies, including mobile applications, messaging services, and social media. Generative and conversational tools, such as ChatGPT, Dress-COV, and triage chatbots, demonstrated effectiveness in expanding access to information, personalizing educational interactions, and supporting health literacy processes.

With respect to target audiences, the studies covered four broad groups: health professionals and managers; students and educators; communities and citizens; and specific populations, such as vaccine-hesitant youth and public health workers. Educational approaches ranged from simulated training and remote teaching, aimed at developing digital competencies, to automated and interactive messaging designed to promote awareness and adherence to preventive measures.

Taken together, the studies highlight the potential of AI to optimize learning, personalize content, improve access to high-quality information, and expand the reach of educational initiatives, especially in contexts with restrictions on in-person activities. A strong integration between AI and digital health was also observed, with applications that extend beyond educational environments to emergency management, participatory surveillance, and community engagement.

On the other hand, the authors point to recurring limitations, such as methodological heterogeneity, absence of educational impact metrics, platform bias, sample constraints, and dependence on specific technological infrastructure. These factors still hinder the comparability and generalizability of findings across the different contexts and studies analyzed.

Table 2 illustrates the breadth of AI-mediated educational strategies applied to the preparedness, response, and recovery phases of public health emergencies, reflecting significant advances in the integration of digital technologies with innovative educational practices. A total of 31 studies were identified that explored different AI modalities, with particular emphasis on chatbots, adaptive learning platforms, ML models, natural language processing (NLP), and generative systems. These technologies were used in multiple contexts and for diverse purposes, ranging from the curation and recommendation of trustworthy content to support for clinical training, participatory education, and health communication.

**Table 2:** AI-based educational strategies: Modality, purpose, platform, and curation (*n* = 31), Brazil, 2025.

ID (AUTHOR, YEAR)	AI MODALITY/STRATEGY	EDUCATIONAL PURPOSE (ESSENCE)	CHANNEL/PLATFORM	SUPERVISION	EQUITY/LANGUAGES
Guo et al. (2024) [8]	ML + NLP for video curation	Filter and recommend trustworthy videos to strengthen health literacy and reduce misinformation	YouTube; apps; messaging	Yes (expert review)	Multilingual potential; integration with official channels
Xie et al. (2024) [9]	AI chatbots (integrative review)	Personalized clinical tutoring/learning in the post-pandemic period	Chatbots/web	Recommended	—
Franchini et al. (2021) [10]	Community chatbot (Dress-COV)	Triage plus participatory education and reinforcement of self-care	Telegram	Yes (curation)	Accessible; community inclusion
Văduva et al. (2023) [11]	eHealth/mHealth/telehealth (with AI)	Remote training and adoption of digital technologies	Apps/telehealth	—	—
Parums (2021) [12]	Editorial (AI in digital health)	Emphasizes informing/training for safe use of technologies	—	—	—
Abdelouahed et al. (2025) [1]	Adaptive AI; intelligent simulators	Continuous training and profile-based personalized content	Educational platforms	Desirable	—
Tekinay (2023) [13]	ChatGPT	Answer public questions in plain language	Web/messaging	—	—
Sezgin and Kocaballi (2025) [14]	Generative AI in messaging	Educational support; assess clarity and relevance of responses	WhatsApp/SMS	Recommended	—
McKee et al. (2025) [15]	Data + AI (applied review)	Segmented communication and decision support in public health	Multiple	—	—
Haupt et al. (2024) [16]	Prompting (role-playing game) in LLM	Media literacy and misinformation detection	Training environments	—	—
Tanui et al. (2024) [17]	Apps with multilingual AI	Inclusive, scalable education in African public health settings	Apps	—	Local languages
Bharel et al. (2024) [18]	Generative AI (perspective)	Support communication, productivity, and insights	Public health agencies	—	Equity/ethics emphasized
Meo et al. (2023) [19]	ChatGPT (performance evaluation)	Complementary study/FAQ tool	Web	—	—
Zeeb et al. (2023) [20]	Apps/digital platforms	Awareness through apps	Corona Health app	—	—
Towler et al. (2023) [21]	ML (topic analysis)	Accelerate insights to guide campaigns	Text data analysis environments	—	—
Ma et al. (2023) [22]	Digital health curriculum (with AI)	Curricular integration and simulated practice	Distance/hybrid education	Faculty/tutors	—
Jia et al. (2023) [23]	AI in surveillance (review)	Train professionals and issue real-time alerts	Surveillance platforms	Institutional	—
He et al. (2022) [24]	AI in imaging (CT)	Educational track for clinical AI use	Imaging services	Professional	—
Grüne et al. (2022) [25]	Symptom diaries + ML	Real-time educational feedback and self-care	Symptom apps	—	—
Weeks et al. (2022) [26]	Personalized vaccine chatbot	Empathic messages to reduce hesitancy	Messaging/chatbot	Content curation	—
Dzau et al. (2022) [27]	Frameworks with AI	Simulations and continuing education	Online platforms	—	—
Wang et al. (2023) [2]	Big-data AI; intelligent tutoring; virtual simulation	Integrate AI into public health curriculum and build AI-literate, emergency-ready professionals	University public health courses; computer-assisted and online learning	Teacher-led; faculty control of AI tools	No explicit equity or multilingual strategy mentioned
Wen et al. (2023) [28]	Bibliometrics (AI/digital)	Map trends to guide education/management	—	—	—
Wang and Li, (2024) [3]	Personalized learning algorithms; predictive analytics; AI-driven simulations	Personalize public health training and support data-informed decision-making	Digital learning platforms; simulation/VR; AI-enhanced online courses	Educator/institutional oversight; emphasis on ethical governance	Discusses fairness and bias; no concrete language/localization plan
Scott and Coiera (2020) [29]	NLP/early warning; modeling	Support messaging and rapid response	Media/reports	—	–
Uohara et al. (2020) [30]	Triage chatbots; ML for research	Scaled recommendations and recruitment	Web/telehealth/virtual ICU	Human curation	—
Montenegro-López (2020) [31]	National app + AI committee	Guidance and local management with user feedback	CoronApp (Colombia)	Technical committee	—
Simsek and Kantarci (2020) [32]	SOFM (optimized mobilization)	Inform logistical planning/education	Models/decision-support tools	—	—
McKillop et al. (2021) [33]	Watson Assistant (chatbots)	COVID-19 information based on CDC/WHO	Watson Assistant chatbots	Documentary curation	Multilingual support
Verma et al. (2025) [34]	YOLO-V5 + 3D distance	Education/compliance with NPIs in hospital environments	CCTV + IEC campaigns (information, education, communication)	Local management	—
Bynon Neely et al. (2024) [35]	ChatGPT for educational SEO	Expand reach/discovery of health videos	YouTube	—	—
Guo et al. (2024) [8]	ML + NLP (detailed pipeline)	Preselect relevant and comprehensible videos	YouTube	Yes (experts)	—

Among the tools analyzed, chatbots and conversational assistants stood out as the most frequently used, appearing in roughly half of the studies. Applications such as Dress-COV, Watson Assistant, and personalized vaccine chatbots were widely employed for interactive education, automated triage, and reduction of vaccine hesitancy, showing positive results in terms of accessibility, communicative empathy, and adherence to preventive measures. In addition, platforms based on ML and NLP, such as those proposed by Guo et al. [8] and Towler et al. [21], were applied to the curation of educational videos and automated topic analysis, contributing to the dissemination of high-quality information and to combating health misinformation.

Adaptive learning systems and intelligent simulators demonstrated strong potential for personalized learning, tailoring content to users’ profiles and individual needs and promoting autonomy, engagement, and educational continuity. These solutions were predominantly implemented in formal learning environments, such as universities and distance education platforms, generally under instructional or technical supervision. AI models integrated into telemedicine and mobile health (mHealth) further expanded opportunities for remote education and home-based support, particularly in low-connectivity settings, helping to reduce digital inequalities.

With regard to channels and platforms, instant messaging services (such as Telegram, WhatsApp, and SMS), mobile applications, web platforms, and hybrid learning environments predominated, underscoring the versatility of AI for large-scale communication and capacity building. Although not all studies reported direct human supervision, expert- or technical committee-led content curation was identified as an essential good practice to ensure accuracy, reliability, and ethical standards in the dissemination of information.

Table 3 synthesizes the distribution of educational functions mediated by AI across the preparedness, response, and recovery phases of public health emergencies, highlighting the versatility and reach of these technologies in different educational and operational contexts. Among the 30 studies analyzed, applications were predominantly concentrated in the preparedness (36%) and response (52%) phases, with fewer initiatives focused on recovery (12%), a trend that reflects the global emphasis on readiness, mitigation, and immediate response during critical periods.

**Table 3:** Educational functions by phase of the pandemic cycle, AI mechanisms, scalability, and gaps (*n* = 31), Brazil, 2025.

ID (AUTHOR, YEAR)	MAIN EDUCATIONAL GOAL	PHASE (PREP/RESPONSE/RECOVERY)	TARGET AUDIENCE	PEDAGOGICAL MECHANISM WITH AI	CHANNEL	SUPERVISION (WHO)	SCALABILITY/OPERATION	LEVEL OF EVIDENCE	KEY GAPS
Guo et al. (2024) [8]	Curation of trustworthy content (literacy)	Response	General public/professionals	ML/NLP for selection; multimedia delivery	YouTube, apps, messaging	Experts (review)	High: integrates with official channels	Methodological (development + evaluation)	Assess behavioral effect; platform dependence
Xie et al. (2024) [9]	Tutoring/educational support post-pandemic	Recovery	Students/educators	Tutor chatbot (personalization)	Web/chatbot	Recommended	High: low marginal cost	Integrative review	Heterogeneity; no clinical outcomes
Franchini et al. (2021) [10]	Triage plus participatory community education	Response	Adults (Telegram)	Validated messages plus reinforcement	Telegram	Curation	High: large-scale app	Implementation study (mixed methods)	Nonequivalent control group
Văduva et al. (2023) [11]	Digital capacity building for nurses	Recovery	Nurses	eHealth/mHealth/telehealth with AI support	Apps/telehealth	NR	Variable: depends on infrastructure	Narrative review	Small sample; no effect measurement
Abdelouahed et al. (2025) [1]	Continuous training and adaptive content	Preparedness	Professionals/managers	Adaptive AI; simulators	Educational platforms	Desirable (faculty/preceptors)	High: online modules	Exploratory qualitative study	No standardized measures
Tekinay (2023) [13]	Public FAQ in plain language	Response	General population	LLM (ChatGPT) Q&A	Web/messaging	NR	High: widely available	Exploratory study	Model bias; update issues
Sezgin and Kocaballi (2025) [14]	Clarity and relevance of conversational responses	Response	Public health FAQs	Generative AI in messaging	WhatsApp/SMS	Recommended	High: ubiquitous channels	Exploratory study	No behavioral outcomes
McKee et al. (2025) [15]	Data-driven segmented communication	Preparedness/response	Public health professionals	Modeling and analytics	Multiple channels	NR	High: policy-informing	Applied review	No field data
Haupt et al. (2024) [16]	Media literacy (misinformation detection)	Preparedness/response	Users/trainees	Role-playing game prompting in LLM	Training environments	NR	High: low cost	Experimental (lab)	Limited sample and scope
Tanui et al. (2024) [17]	Inclusive multilingual education	Preparedness/response	African populations	Apps with AI (local languages)	Apps	NR	High: scalable	Narrative review	Descriptive evidence only
Bharel et al. (2024) [18]	Institutional communication and productivity	Preparedness	Public health agencies/professionals	Generative AI for summarization/generation	Institutional platforms	NR	Moderate: requires governance	Perspective	No empirical data
Meo et al. (2023) [19]	Study/educational FAQ	Preparedness/response	Students/professionals	LLM (Q&A)	Web	NR	High	Performance evaluation	No link to behavior
Zeeb et al. (2023) [20]	Awareness through regional apps	Response	Population (Bremen)	Apps plus algorithms	Corona Health app	NR	Moderate: local context	Descriptive narrative	No causal evaluation
Towler et al. (2023) [21]	Rapid insights from qualitative data	Response	Public health teams	ML (topic modeling) for rapid synthesis	Analytic environments	NR	High: accelerates decision-making	Methodological study	Loss of cultural nuances
Ma et al. (2023) [22]	Integration of digital health/AI into curricula	Preparedness	Health students	Distance/hybrid learning with AI	Academic environment	Faculty	High: institutional	Cross-sectional study	Self-reported data; no impact outcomes
Jia et al. (2023) [23]	Surveillance training and alerts	Preparedness/response	Professionals/public	AI for detection and alerts	Surveillance platforms	Institutional	High	Narrative review	No primary data
He et al. (2022) [24]	Training for clinical AI use (imaging)	Response	Health professionals	AI diagnostic assistance	Imaging services	Professional	Moderate: requires infrastructure	Observational study	Retrospective data; variability
Grüne et al. (2022) [25]	Self-care via symptom feedback	Response	App users	ML in symptom diaries	Apps	NR	High	Retrospective observational study	Self-report; external validation
Weeks (2022) [26]	Reduction of vaccine hesitancy	Response	Urban youth	Personalized chatbot (empathic messages)	Messaging/chatbot	Curation	High: messaging	Qualitative study	Limited generalizability
Dzau et al. (2022) [27]	Digital training and simulations	Preparedness	Professionals/students	Simulations and remote teaching	Online platforms	NR	High	Narrative review	No impact data
Wang et al. (2023) [2]	Modernize public health curriculum and train emergency-ready professionals	Preparedness	Public health students; public health educators	AI-based intelligent tutoring; data-driven curriculum design; computer-assisted learning using big data	University courses; online/computer-assisted	Teachers/faculty	Potentially high via e-learning; only proposed	Narrative review	No implementation; no learning outcomes
Wen et al. (2023) [28]	Mapping thematic frontiers	Preparedness	—	Bibliometrics of digital/AI	—	NR	High: guides agendas	Bibliometric study	Coverage and language bias
Wang and Li (2024) [3]	Personalize and modernize public health training	Response	Public health/medical students; health professionals	Adaptive learning; AI simulations; analytics	Digital platforms; online courses; simulation	Educators/institutions	High theoretical scalability; not tested	Narrative perspective	No primary data; few concrete models for LMICs
Scott and Coiera (2020) [29]	Data-informed messaging and rapid response	Response	Patients/professionals	NLP/early warning; modeling	Media/reports	NR	High	Critical narrative review	No educational evaluation
Uohara et al. (2020) [30]	Scaled recommendations and recruitment	Response	Professionals/public	Triage chatbots; ML for research	Web/telehealth/virtual ICU	Human curation	High	Narrative review	Governance and consent
Montenegro-López (2020) [31]	Guidance and local management	Response	Professionals/patients	National app plus AI committee	CoronApp	Technical committee	High: national level	Descriptive study	Validation of decision rules; asymptomatic cases
Simsek and Kantarci (2020) [32]	Mobilization planning and logistics	Preparedness	Managers	SOFM for optimal routes	Models/decision-support tools	NR	High: simulation-based	Case/modeling study	Dependence on assumptions
McKillop et al. (2021) [33]	Automated informational service	Response	Citizens	Chatbots (Watson Assistant)	Web/chatbots	Documentary curation	High	Mixed-methods exploratory study	No metrics for satisfaction, time, or cost
Verma et al. (2025) [34]	Compliance with NPIs in hospitals	Response	Visitors/patients	Video detection (YOLO-V5 + 3D)	CCTV + IEC campaigns	Local management	Moderate: hardware-dependent	Feasibility study (mixed methods)	No control group; confounding factors
Bynon Neely et al. (2024) [35]	Multimedia educational reach	Response	Communities/public health workers	YouTube + SEO (ChatGPT)	YouTube	NR	High: low cost	Exploratory study	History of misinformation

*Notes:* Phases—preparedness (prep), response (response), and recovery (recovery). NR = not reported. “Level of evidence” refers to the type of study/report.

In the preparedness phase, AI was widely used in professional training processes and in the integration of digital health into curricula through intelligent simulations, adaptive learning systems, and instructional platforms. These approaches supported the development of technical and digital competencies, preparing professionals and students to work in scenarios characterized by risk, uncertainty, and information overload. In addition, solutions based on modeling and predictive analytics were applied to optimize resource management, logistical planning, and institutional communication.

In the response phase, most of the experiences described centered particularly on the use of chatbots, NLP systems, ML models, and multimedia platforms. Tools such as Watson Assistant, Dress-COV, and national applications like CoronApp were widely employed for participatory education, automated triage, reduction of vaccine hesitancy, and media literacy, standing out for their high scalability and low operational cost. In surveillance and risk communication contexts, AI was also applied to the curation of trustworthy content, monitoring of misinformation, and provision of personalized conversational responses, expanding the reach of educational messages and strengthening community engagement.

In the recovery phase, studies focused on initiatives for digital capacity building and psychosocial-educational support in the post-pandemic period, with emphasis on conversational tutoring models, eHealth/mHealth, and AI-assisted distance education aimed at professional requalification and the resumption of academic activities. These experiences demonstrated the potential of AI to ensure educational continuity and reduce inequalities in access, although they still rely heavily on technological infrastructure and regional connectivity.

With regard to scalability, more than 70% of the experiences analyzed showed operational feasibility at scale, mainly through web platforms and mobile messaging services, which enabled broad dissemination of information and dynamic interaction with diverse audiences. However, important gaps remain, such as methodological heterogeneity, the absence of educational impact metrics, dependence on commercial platforms, and the lack of behavioral and clinical indicators that would allow assessment of the actual effects on learning and practice change.

**Figure 1:**
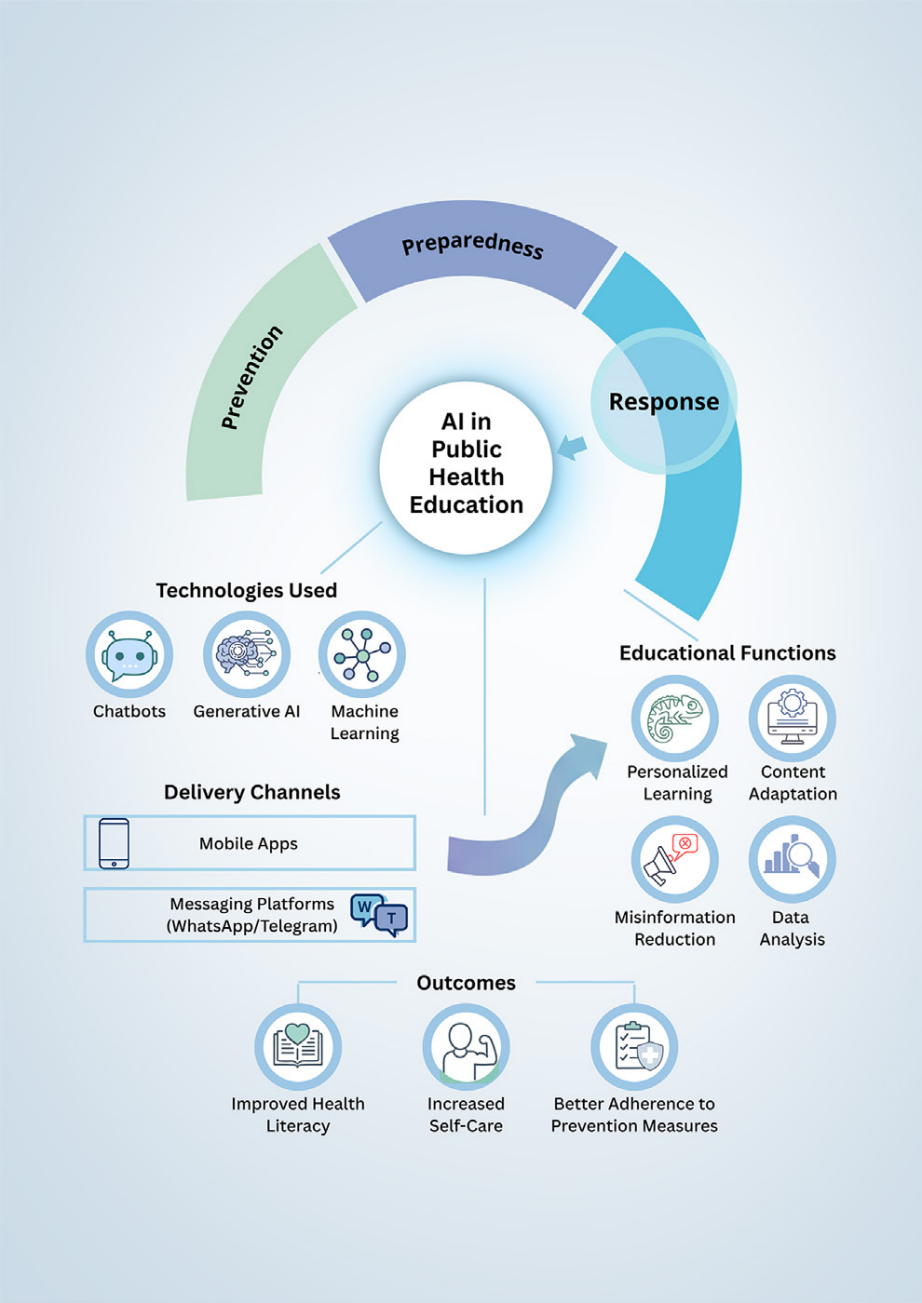
AI framework for public health education and emergency response.

Figure 1 presents a conceptual model that illustrates the applications of AI in public health education across the pandemic preparedness and response cycle. The figure shows that AI technologies, especially chatbots, generative models, and ML systems, have been used predominantly in the response phase of health emergencies. These tools are delivered through mobile applications, web platforms, and messaging services (such as WhatsApp and Telegram), enabling broad dissemination of educational content. Their main educational functions include personalization of learning, content adaptation, risk communication, reduction of misinformation, and rapid data analysis. Together, these AI-mediated strategies contribute to improving health literacy, increasing self-care, and promoting greater adherence to preventive measures among the population.

## Discussion

### Role of artificial intelligence in public health education across pandemic phases

This study mapped evidence on the use of AI-based technologies in educational strategies aimed at planning the preparedness, response, and recovery stages of public health emergencies. The results of the studies assessed in this review reveal the consolidation of AI as a health education tool in the context examined, enabling risk identification, optimization of responses, and personalization of educational content for health professionals and the general public. These findings are corroborated by a previous study that explored lessons learned during the COVID-19 pandemic and offered insights into the use of AI as support in crisis scenarios [1].

Public health emergencies constitute serious threats to population health, as they can result in widespread dissemination of infectious agents, high morbidity and mortality, and substantial impacts on the reorganization of health services [36]. In this context, AI emerges as a strategic technology by supporting rapid, data-driven decision-making, enhancing risk identification, and guiding timely interventions in epidemic and pandemic events. However, despite its considerable potential, the effectiveness of AI solutions depends on structural factors, such as global collaboration, robust governance, compliance with ethical principles, standardization and validation of information, interoperability between systems, and assurance of equity in access to and use of technologies [1, 37, 38].

The literature indicates that AI has substantial potential as an educational resource and as support for actions across all stages of pandemic and epidemic management. In the preparedness phase, algorithms applied to the analysis of large datasets can be used to forecast outbreaks, inform public policy formulation, and support vaccine development [1, 38]. During the response phase, AI can optimize healthcare delivery through efficient resource management, risk stratification, and reduction of health workers’ exposure to infectious agents [15, 38]. In the recovery phase, these technologies contribute to impact evaluation, monitoring of clinical outcomes, and the promotion of continuing health education [15].

Among the AI systems identified in this review, there was a predominance of chatbots and generative models (such as ChatGPT, Dress-COV, and Watson Assistant), underscoring the consolidation of conversational AI as a strategic tool for risk communication, triage, and health education. In addition to expanding access to information, these solutions can provide personalized guidance for behavior change, tailoring communication to users’ levels of digital literacy and promoting greater engagement.

However, the studies analyzed emphasize that, for their potential to be fully realized, such technologies must be developed and continuously refined with a focus on equity, accountability, and cultural sensitivity, ensuring reliability and fairness in their application within public health contexts [14].

Adaptive learning systems and intelligent simulators demonstrate high potential, particularly in the preparedness phase, by enabling personalized training and continuous learning, thereby contributing to the development of essential digital competencies for working in emergency situations [1]. Evidence indicates that these technologies adjust content according to individual performance, simulate realistic clinical scenarios, and monitor users’ progress, making teaching more efficient, accessible, and learner-centered, including at scale, with a positive impact on timely response capacity during health crises [1].

In addition, in the recovery phase, such tools can support ongoing workforce development, enabling feedback loops in learning processes and the incorporation of post-event lessons. Nevertheless, despite the substantial benefits, close integration between AI solutions and human supervision remains essential to ensure pedagogical quality, equity, and adherence to ethical principles, especially in high-pressure contexts and situations involving critical decision-making [38].

### Structural imbalance across pandemic phases and implications for health system resilience

The evidence analyzed in this review indicates that AI has been used predominantly in the response phase of public health emergencies, with more limited application in the preparedness and recovery phases. During health crises, the demand for rapid and accurate decisions drives the use of automated technologies for data generation and analysis, resource allocation, triage, and decision support [39, 40]. By contrast, the preparedness and recovery phases require long-term planning, systems integration, and sustained investment, elements that have historically received lower priority and funding from decision-makers [40]. Overcoming this imbalance is essential for strengthening resilient health systems capable of anticipating risks, reducing vulnerabilities, and incorporating post-crisis learning [32, 40].

This concentration of AI-enabled educational initiatives in the response phase represents a structural limitation for health system resilience. When educational uses of AI are primarily activated during crises, they tend to function as short-term, reactive tools rather than as components of sustained capacity building. Insufficient integration of AI-based education into the preparedness phase limits the development of digital literacy, critical appraisal skills, and institutional familiarity with these technologies before emergencies occur. Likewise, the relative neglect of the recovery phase constrains opportunities for systematic learning, workforce requalification, and the incorporation of lessons learned into future training cycles. As a result, health systems risk reproducing recurrent patterns of vulnerability, entering successive emergencies without consolidated educational infrastructures capable of supporting anticipatory, adaptive, and equitable responses.

### Evidence gaps, equity, and governance challenges in AI-mediated education

With regard to educational evidence, the included studies exhibit considerable methodological heterogeneity and, in most cases, lack robust indicators for assessing the impact of AI on learning, behavior change, or improvement in clinical practice. A critical gap identified across the reviewed studies is the absence of standardized and validated metrics to assess educational outcomes of AI-mediated interventions. Most initiatives rely on proxy indicators such as engagement, user satisfaction, or self-reported knowledge gains, which limits comparability across settings and impedes cumulative evidence generation. Without agreed-upon frameworks to measure competencies, skill acquisition, or changes in professional practice, it remains difficult to determine whether AI-based educational tools produce meaningful and sustained impacts beyond immediate crisis communication [1, 22, 41, 39].

Equally underexplored are dimensions related to equity, cost-effectiveness, and long-term sustainability. Few studies explicitly examined whether AI-enabled educational strategies reduce or exacerbate existing social and digital inequalities, particularly in low-resource or marginalized contexts. Moreover, economic evaluations were rarely reported, and operational sustainability beyond emergency funding cycles remained unclear. This lack of analysis constrains the translation of promising pilot initiatives into scalable and durable public health education policies [17, 18, 22, 42, 43].

The review also identified a scarcity of validated instruments specifically designed to measure the effect of AI-mediated educational interventions in pandemic contexts [41]. In this context, the literature recommends the development of metrics that capture not only engagement or knowledge gains, but also competency acquisition, patient safety, and health outcomes [1].

Finally, important gaps persist in governance models and ethical validation processes for AI-mediated educational content. Although several studies acknowledged the need for human oversight, detailed descriptions of accountability mechanisms, validation workflows, data governance, and bias mitigation strategies were uncommon. The limited transparency surrounding these processes raises concerns regarding reliability, trust, and ethical compliance, particularly when educational interventions are deployed at scale during high-stakes public health emergencies [3, 14, 18, 37, 39].

The studies analyzed also revealed a strong dependence on commercial platforms and limited transparency regarding ethical aspects and human validation processes for AI-mediated educational content, underscoring the need for coordinated action at technical, institutional, regulatory, and societal levels to ensure governance, reliability, and accountability in the application of educational algorithms [39]. Although AI-mediated education has considerable potential to mitigate digital inequalities during pandemics and other public health emergencies, by expanding access to knowledge and supporting the inclusion of diverse audiences, its implementation is still constrained by structural barriers such as insufficient technological infrastructure, shortages of qualified professionals, data limitations, and ethical challenges related to privacy and information security [42].

In this regard, the responsible advancement of these technologies requires recognition and mitigation of inequalities that may be reproduced or exacerbated by the inappropriate use of AI. For digital solutions to be genuinely inclusive, it is essential to incorporate digital determinants of health, such as usability, accessibility, interactivity, digital literacy, and technological availability, into their design, in close alignment with the social determinants of health, which shape opportunities for access, learning, and engagement with digital technologies [43]. Thus, the integration of these determinants is a fundamental requirement for promoting equity in access to digital health and strengthening health justice across different populations.

The establishment of strategic alliances among academic institutions, health services, the technology industry, and regulatory bodies demands ethical governance, system interoperability, open infrastructure, ongoing professional training, and collaborative research [3, 38]. Such efforts are essential to ensure that AI applied to health education is accessible, safe, and capable of responding appropriately to the global challenges posed by public health emergencies.

### Limitations

This scoping review has some limitations that should be acknowledged. As a mapping review, it aimed to describe the breadth and characteristics of the available evidence rather than to assess effectiveness or causal relationships, and no formal risk-of-bias assessment was conducted. The heterogeneity of study designs, interventions, and reported outcomes limited direct comparison across studies. In addition, the reliance on published literature and selected gray literature may have resulted in the omission of unpublished initiatives or rapidly evolving implementations, particularly those developed during acute phases of public health emergencies. Finally, most included studies focused on high- and middle-income settings, which may limit the generalizability of the findings to low-resource contexts.

## Conclusion

The integration of AI into public health education represents a strategic innovation with the potential to improve social and professional responses to pandemics and other health emergencies by personalizing learning, expanding the reach of information, and supporting evidence-based decision-making. However, this review found that such integration remains largely concentrated in the response phase, highlighting the need for greater investment in structural preparedness approaches and in the continuity of educational efforts in the post-crisis period.

For AI to be fully effective, it is necessary to develop standardized metrics that assess not only knowledge acquisition but also behavior change and impacts on care, to expand technological infrastructure and strengthen the digital competencies of the population and health workers, and to consolidate ethical governance, interoperability, and algorithmic transparency. In this sense, AI emerges as a crucial element for strengthening resilient health systems and promoting faster, more equitable, and more effective responses to future events of global public health relevance.

## Data Availability

All data generated or analyzed during this study are included in the published articles reviewed and their supplementary materials. As this is a scoping review based exclusively on publicly available literature, no new datasets were generated.
